# Inhibition of LRRK2 kinase activity leads to dephosphorylation of Ser^910^/Ser^935^, disruption of 14-3-3 binding and altered cytoplasmic localization

**DOI:** 10.1042/BJ20100784

**Published:** 2010-08-27

**Authors:** Nicolas Dzamko, Maria Deak, Faycal Hentati, Alastair D. Reith, Alan R. Prescott, Dario R. Alessi, R. Jeremy Nichols

**Affiliations:** *MRC Protein Phosphorylation Unit, College of Life Sciences, University of Dundee, Dow Street, Dundee DD1 5EH, Scotland, U.K.; †Department of Neurology, Institut National de Neurologie, Tunis, Tunisia; ‡External Alliances & Development, GlaxoSmithKline (China) R&D Co. Ltd, Medicines Research Centre, Gunnels Wood Road, Stevenage SG1 ZNY U.K.; §Division of Cell Biology and Immunology, College of Life Sciences, University of Dundee, Dow Street, Dundee DD1 5EH, Scotland, U.K.

**Keywords:** cell-based assay, drug discovery, 14-3-3 protein kinase inhibitor, leucine-rich repeat protein kinase 2 (LRRK2), Parkinson's disease, protein phosphorylation, DIG, digoxigenin, DMEM, Dulbecco's modified Eagle's medium, EBV, Epstein–Barr virus, ERK, extracellular-signal-regulated kinase, FBS, fetal bovine serum, GFP, green fluorescent protein, HEK, human embryonic kidney, HRP, horseradish peroxidase, JNK, c-Jun N-terminal kinase, KLH, keyhole-limpet haemocyanin, LRRK2, leucine-rich repeat protein kinase 2, MAPK, mitogen-activated protein kinase, MEK, MAPK/ERK kinase, mTOR, mammalian target of rapamycin, MYPT, myosin phosphatase-targeting, NP-40, Nonidet P40, PFA, paraformaldehyde, PI3K, phosphoinositide 3-kinase, ROCK, Rho-kinase, TBST, Tris-buffered saline with Tween 20

## Abstract

LRRK2 (leucine-rich repeat protein kinase 2) is mutated in a significant number of Parkinson's disease patients. Since a common mutation that replaces Gly^2019^ with a serine residue enhances kinase catalytic activity, small-molecule LRRK2 inhibitors might have utility in treating Parkinson's disease. However, the effectiveness of inhibitors is difficult to assess, as no physiological substrates or downstream effectors have been identified that could be exploited to develop a robust cell-based assay. We recently established that LRRK2 bound 14-3-3 protein isoforms via its phosphorylation of Ser^910^ and Ser^935^. In the present study we show that treatment of Swiss 3T3 cells or lymphoblastoid cells derived from control or a Parkinson's disease patient harbouring a homozygous LRRK2(G2019S) mutation with two structurally unrelated inhibitors of LRRK2 (H-1152 or sunitinib) induced dephosphorylation of endogenous LRRK2 at Ser^910^ and Ser^935^, thereby disrupting 14-3-3 interaction. Our results suggest that H-1152 and sunitinib induce dephosphorylation of Ser^910^ and Ser^935^ by inhibiting LRRK2 kinase activity, as these compounds failed to induce significant dephosphorylation of a drug-resistant LRRK2(A2016T) mutant. Moreover, consistent with the finding that non-14-3-3-binding mutants of LRRK2 accumulated within discrete cytoplasmic pools resembling inclusion bodies, we observed that H-1152 causes LRRK2 to accumulate within inclusion bodies. These findings indicate that dephosphorylation of Ser^910^/Ser^935^, disruption of 14-3-3 binding and/or monitoring LRRK2 cytoplasmic localization can be used as an assay to assess the relative activity of LRRK2 inhibitors *in vivo*. These results will aid the elaboration and evaluation of LRRK2 inhibitors. They will also stimulate further research to understand how phosphorylation of Ser^910^ and Ser^935^ is controlled by LRRK2, and establish any relationship to development of Parkinson's disease.

## INTRODUCTION

Autosomal dominant missense mutations within the gene encoding LRRK2 (leucine-rich repeat protein kinase 2) predispose humans to Parkinson's disease [1,2]. Patients with LRRK2 mutations generally develop Parkinson's disease with clinical appearance and symptoms indistinguishable from idiopathic disease at around 60–70 years of age [3]. Mutations in LRRK2 account for 4% of familial Parkinson's disease, and are observed in 1% of sporadic Parkinson's disease patients [3]. Over 40 missense mutations have been reported [4]. The most frequent mutation comprises an amino acid substitution of the highly conserved Gly^2019^, located within the subdomain VII-DFG motif of the kinase domain for a serine residue [4], which enhances the protein kinase activity of LRRK2 approx. 2-fold [5]. Two other mutations (R1728H and T2031S) also increase LRRK2 protein kinase activity [6]. These observations indicate that inhibitors of LRRK2 may have utility for the treatment of Parkinson's disease.

The intrinsic protein kinase catalytic activity of LRRK2 is readily measured *in vitro* using assays employing peptide substrates such as LRRKtide (RLGRDKYKTLRQIRQGNTKQR) [7] or Nictide [8] (RLGWWRFYTLRRARQGNTKQR). This has made it possible to undertake screens to identify inhibitors. Recent work has shown that a widely deployed ROCK (Rho-kinase) inhibitor termed H-1152 also inhibited LRRK2 with similar potency (IC_50_ of 150 nM) [8]. The multi-target tyrosine kinase inhibitor sunitinib, used for the treatment of renal cell carcinoma and other cancers, also inhibits LRRK2 (IC_50_ of 20 nM) [8–10]. We have also found that the structurally diverse H-1152 and sunitinib inhibitors suppress the activity of the LRRK2(G2019S) mutant 2–4-fold more potently than wild-type LRRK2 [8]. On the basis of molecular modelling of the LRRK2 kinase domain we have previously designed a drug-resistant LRRK2(A2016T) mutant that is normally active, but 32-fold less sensitive to H-1152 and 12-fold less sensitive to sunitinib [8].

A bottleneck in the development of LRRK2 inhibitors is how to assess the relative effectiveness of these compounds *in vivo*, as little is known about how LRRK2 is regulated and what its substrates are. We recently demonstrated that LRRK2 interacts with 14-3-3 protein isoforms and binding is mediated through phosphorylation of two conserved LRRK2 residues (Ser^910^ and Ser^935^) that lie before the leucine-rich repeats [6]. Disruption of 14-3-3 interaction by mutation of Ser^910^ and/or Ser^935^ to an alanine residue does not affect LRRK2 kinase activity but has an impact on LRRK2 localization, causing it to accumulate within cytoplasmic pools instead of being diffusely localized throughout the cytoplasm [6]. It has also been reported that various mutant forms, such as LRRK2(R1441C) and LRRK2(Y1699C), accumulated within cytosolic pools that were suggested to comprise aggregates of misfolded protein [11]. By comparing the properties of 41 Parkinson's-disease-associated mutations of LRRK2 we found that LRRK2(R1441C) and LRRK2(Y1699C), as well as eight other mutants, displayed reduced phosphorylation of Ser^910^/Ser^935^ and interaction with 14-3-3 [6]. Consistent with 14-3-3 regulating localization, most mutants displaying reduced 14-3-3 binding accumulated within cytoplasmic pools [6]. Although a great deal of further analysis is required to work out how 14-3-3 binding regulates LRRK2, these results suggest that disrupting the interaction of LRRK2 with 14-3-3 could be linked to Parkinson's disease.

In the present study we provide pharmacological evidence that LRRK2 protein kinase activity indirectly regulates phosphorylation of Ser^910^ and Ser^935^ and hence controls binding to 14-3-3 isoforms. Thus inhibition of LRRK2 causes dephosphorylation of Ser^910^/Ser^935^ and hence dissociation of 14-3-3 binding causing LRRK2 to accumulate within cytoplasmic pools. Our study indicates that monitoring phosphorylation of Ser^910^/Ser^935^, 14-3-3 binding and/or visualization of LRRK2 accumulation of LRRK2 into cytoplasmic pools can be deployed to monitor the activity of LRRK2 inhibitors in cells.

## MATERIALS AND METHODS

### Reagents and general methods

Tissue-culture reagents were from Life Technologies and [γ^32^P]ATP was from PerkinElmer. P81 phosphocellulose paper was from Whatman. Pepceuticals synthesized Nictide. The Flp-in T-REx system was from Invitrogen and stable cell lines, generated according to the manufacturer's instructions by selection with hygromycin, have been described previously [8]. Restriction enzyme digests, DNA ligations and other recombinant DNA procedures were performed using standard protocols. All mutagenesis was carried out using the QuikChange™ site-directed mutagenesis kit (Stratagene). DNA constructs used for transfection were purified from *Escherichia coli* DH5α using Qiagen or Invitrogen plasmid Maxi kits according to the manufacturer's protocol. All DNA constructs were verified by DNA sequencing, which was performed by the Sequencing Service, School of Life Sciences, University of Dundee, Scotland, U.K., using DYEnamic ET terminator chemistry (Amersham Biosciences) on Applied Biosystems automated DNA sequencers. H-1152 was purchased from Calbiochem, Sunitinib was from LC Laboratories and generation and use of GSK429286A was described previously [8,12]. Other inhibitors used (in Supplementary Figure S2 at http://www.BiochemJ.org/bj/430/bj4300405add.htm) were obtained from the Division of Signal Transduction Therapy Unit at the University of Dundee.

### Buffers

Lysis buffer contained 50 mM Tris/HCl, pH 7.5, 1 mM EGTA, 1 mM EDTA, 1 mM sodium orthovanadate, 10 mM sodium β-glycerophosphate, 50 mM NaF, 5 mM sodium pyrophosphate, 0.27 M sucrose, 1 mM benzamidine and 2 mM PMSF, and was supplemented with either 1% (v/v) Triton X-100 or 0.5% NP-40 (Nonidet P40) with 150 mM NaCl as indicated. Buffer A contained 50 mM Tris/HCl, pH 7.5, 50 mM NaCl, 0.1 mM EGTA, 0.1% 2-mercaptoethanol and 0.27 M sucrose.

### Cell culture, treatments and cell lysis

HEK (human embryonic kidney)-293 and Swiss 3T3 cells were cultured in DMEM (Dulbecco's modified Eagle's medium) supplemented with 10% (v/v) FBS (fetal bovine serum), 2 mM glutamine and 1× penicillin/streptomycin solution. T-REx cell lines were cultured in DMEM supplemented with 10% (v/v) FBS and 2 mM glutamine, 1× penicillin/streptomycin solution, 15 μg/ml blastocidin and 100 μg/ml hygromycin. Cultures were induced to express the indicated protein by inclusion of 1 μg/ml doxycycline in the culture medium for the indicated times.

Peripheral blood lymphocytes were collected from individuals within an Arab–Berber population, screened for the LRRK2(G2019S) mutation [13] and lymphoblastoid cell lines were generated by EBV (Epstein–Barr virus) transformation of B lymphocytes using standard methods (European Collection of Cell Cultures). Cell-line ANK is derived from a 47-year-old individual homozygous for the LRRK2(G2019S) mutation who presented with Parkinson's disease. Cell-line AHE is derived from a 31-year-old individual, lacking mutation at the LRRK2 Gly^2019^ residue, and presented with no disease. Human lymphoblastoid cells were maintained in RPMI 1640 with 10% FBS, 2 mM glutamine, 1× penicillin/streptomycin solution and were maintained at cell density of 0.3×10^6^–2×10^6^ cells per ml. Cell transfections were performed by the polyethyleneimine method [14]. Where inhibitors were utilized, they were dissolved in DMSO and used at the indicated concentrations with an equivalent volume of DMSO used as a control. The final concentration of DMSO in the culture medium was never more than 0.1%. Inhibitors were added to the culture medium for the indicated times before lysis. For a 15-cm-diameter dish, HEK-293 cells were lysed with 1.0 ml and Swiss 3T3 cells were lysed with 0.6 ml of lysis buffer supplemented with the indicated detergent, and clarified by centrifugation at 16000 ***g*** at 4 °C for 10 min. When not used immediately, all lysate supernatants were snap-frozen in liquid nitrogen and stored at −80 °C until use. Protein concentrations were determined using the Bradford method with BSA as the standard.

### Antibodies

Anti-LRRK2-(100–500) (S348C and S406C) and anti-LRRK2-(2498–2514) (S374C) antibodies were described previously [8]. The antibody against LRRK2 phosphoSer^910^ (S357C) was generated by injection of the KLH (keyhole-limpet haemocyanin)-conjugated phosphopeptide VKKKSNpSISVGEFY (where pS is phosphoserine) into sheep and was affinity purified by positive and negative selection against the phospho- and de-phospho-peptides respectively. The antibody against LRRK2 phosphoSer^935^ (S814C) was generated by injection of the KLH-conjugated phosphopeptide NLQRHSNpSLGPIFDH into sheep and was affinity purified by positive and negative selection against the phospho and de-phospho-peptides respectively. Sheep polyclonal antibody S662B was raised against chicken MBP (maltose-binding protein)–MYPT (myosin phosphatase-targeting) amino acids (714–1004). The rabbit polyclonal antibody against MYPT phosphoThr^850^ was from Upstate Biotechnology (catalogue number 36-003). Anti-GFP (green fluorescent protein) antibody (S268B) was raised against recombinant GFP and affinity purified against the antigen. Anti-FLAG M2 antibody and affinity matrix were from Sigma–Aldrich. Nanotrap GFP-binder affinity matrix was from ChromoTek. The rabbit polyclonal antibody recognizing 14-3-3 (catalogue number K-19) and control anti-(rabbit IgG) antibody (catalogue number SC-2027) were from Santa Cruz Biotechnology.

### Immunological procedures

Cell lysates (10–30 μg) were resolved by SDS/PAGE or Novex 4–12% gradient gels, and electroblotted on to nitrocellulose membranes. Membranes were blocked with 5% (w/v) skimmed milk powder in TBST (Tris-buffered saline with Tween 20) buffer (50 mM Tris/HCl, pH 7.5, 0.15 M NaCl and 0.1% Tween 20). For anti-phospho- antibodies, primary antibody was used at a concentration of 1 μg/ml, diluted in 5% (w/v) skimmed milk powder in TBST with the inclusion of 10 μg/ml dephosphorylated peptide. All other antibodies were used at 1 μg/ml in 5% (w/v) skimmed milk powder in TBST. Detection of immune complexes was performed using either fluorophore-conjugated secondary antibodies (Molecular Probes) followed by visualization using an Odyssey® LI-COR imaging system or by HRP (horseradish peroxidase)-conjugated secondary antibodies (Pierce) and an enhanced chemiluminescence reagent. For immunoprecipitations, antibody was non-covalently coupled to Protein G–Sepharose with of 1 μg of antibody/μl of beads, or anti-FLAG M2–agarose was utilized. Cell lysate was incubated with coupled antibody for 1 h. Immune complexes were washed twice with lysis buffer, supplemented with 0.3 M NaCl, and twice with buffer A. Precipitates were either used as a source of kinase or immediately analysed by immunoblot. DIG (digoxigenin)-labelled 14-3-3 for use in overlay far-Western blotting analysis was prepared as described in [15]. To directly assess 14-3-3 interaction with LRRK2, immunoprecipitates were electroblotted on to nitrocellulose membranes and blocked with 5% (w/v) skimmed milk powder for 30 min. After washing with TBST, membranes were incubated with DIG-labelled 14-3-3 diluted to 1 μg/ml in 5% (w/v) BSA in TBST overnight at 4 °C. DIG 14-3-3 was detected with HRP-labelled anti-DIG Fab fragments (Roche).

### LRRK2 immunoprecipitation kinase assays

Peptide kinase assays were set up in a total volume of 50 μl with immunoprecipitated LRRK2 as a source of kinase, in 50 mM Tris/HCl, pH 7.5, 0.1 mM EGTA, 10 mM MgCl_2_ and 0.1 mM [γ^32^P]ATP (~500–1000 c.p.m./pmol) in the presence of 20 μM Nictide peptide substrate. Reactions were terminated by applying 40 μl of the reaction mixture on to P81 phosphocellulose paper and immersion in 50 mM phosphoric acid. After extensive washing, reaction products were quantified by Cerenkov counting.

### Fluorescence microscopy

HEK-293 Flp-in T-REx were purchased from Invitrogen and stable cells harbouring GFP-tagged wild-type and mutant forms of LRRK2 were generated using standard protocols. Cells were plated in four-well glass-bottomed CC^2^™-coated chamber slides (Nunc). At 1 day after plating, cells were induced with 0.1 μg/ml doxycycline and 24 h later, cells were fixed in 4% (w/v) PFA (paraformaldehyde) in PBS. For staining of microtubules, cells, induced as above, were washed twice in 37 °C medium, and fixed with 4% (w/v) PFA for 15 min. Cells were washed with PBS, permeabilized with 1% (v/v) NP-40 in PBS for 10 min then blocked with 10% (v/v) normal goat serum after washing. Microtubules were stained with rat monoclonal clone YL1/2 (a gift form Dr Inke Nathke, University of Dundee). Cells were stained with goat anti-rat antibody conjugated to Alexa Fluor®594 (Molecular Probes). Cells were mounted in ProLong Gold (Invitrogen) and imaged under the same settings for each mutant, on a Zeiss LSM 700 confocal microscope using an αPlan-Apochromat ×100 objective.

## RESULTS

### LRRK2 inhibitors induced dephosphorylation of Ser^910^/Ser^935^ and disrupted 14-3-3 binding

**Figure 1:**
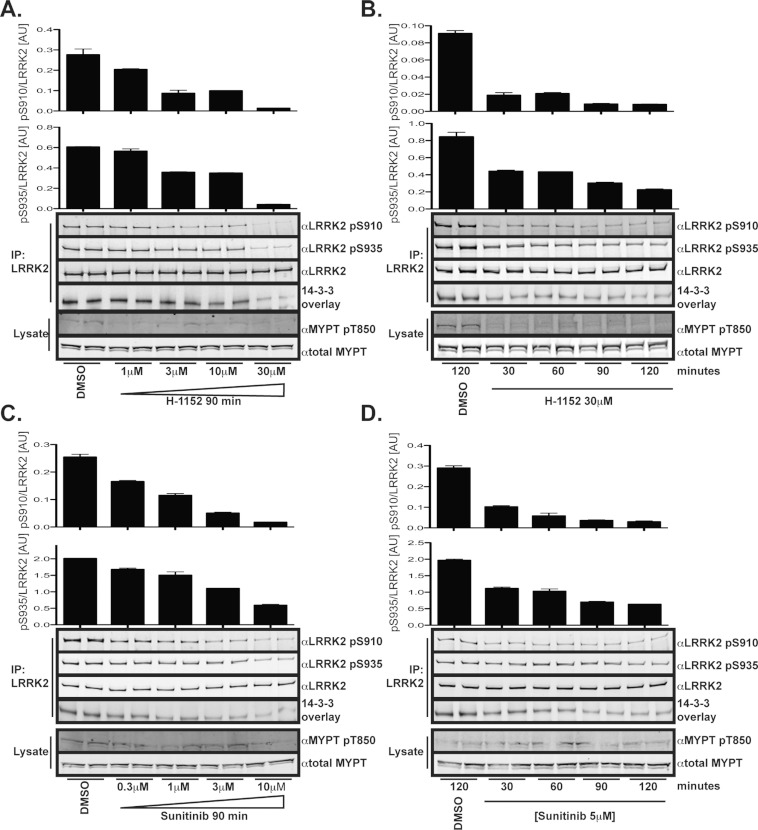
H-1152 and sunitinib treatment leads to dephosphorylation of Ser^910^ and Ser^935^ and disruption of 14-3-3 interaction (**A**) Endogenous LRRK2 was immunoprecipitated (IP) with anti-LRRK2-(100–500) (S348C) antibody from Swiss 3T3 cells treated with DMSO vehicle control or the indicated concentrations of H-1152 for 90 min. Immunoprecipitates were subjected to immunoblot analysis with the indicated antibody as well as 14-3-3 overlay far-Western analysis. The immunoblot analysis was quantified by Odyssey® LI-COR analysis with the histogram at the top of each panel representing the ratio of Ser^910^ phosphorylation to total LRRK2, the lower histogram represents the ratio of Ser^935^ phosphorylation to total LRRK2. (**B**) Endogenous LRRK2 immunoprecipitates were analysed as in (**A**), except that cells were treated with 30 μM H-1152 for the indicated time prior to cell lysis. (**C** and **D**) Endogenous LRRK2 immunoprecipitates were analysed as in (**A** and **B**) respectively, except that sunitinib was employed rather than H-1152. Results are means±S.E.M. and are representative of at least two separate experiments performed in duplicate. AU, arbitrary units.

To investigate how inhibition of LRRK2 protein kinase activity affects on Ser^910^/Ser^935^ phosphorylation, we initially treated Swiss 3T3 cells with increasing amounts of the LRRK2 inhibitors H-1152 (Figure 1A) or sunitinib (Figure 1C). Strikingly, H-1152 and sunitinib induced a dose-dependent dephosphorylation of endogenous LRRK2 at Ser^910^ and Ser^935^, accompanied by a concomitant reduction in 14-3-3 binding. We found that 10–30 μM H-1152 or 3–10 μM sunitinib induced almost complete dephosphorylation of Ser^910^ and Ser^935^, resulting in a loss of 14-3-3 binding. The inhibitory effects of H-1152 (Figure 1B) and sunitinib (Figure 1D) on endogenous LRRK2-Ser^910^/Ser^935^ phosphorylation and 14-3-3 binding were observed within 30 min and were sustained for at least 2 h. H-1152 treatment caused a marked decrease in the phosphorylation of Thr^850^ of the ROCK substrate MYPT1, whereas sunitinib did not (Figure 1), suggesting that the dephosphorylation of LRRK2 at Ser^910^ and Ser^935^ was not due to ROCK inhibition. To further rule out an effect of ROCK in regulating Ser^910^ and Ser^935^ phosphorylation, we employed the selective ROCK inhibitor GSK429286A that does not inhibit LRRK2 [8] and found that this compound did not inhibit Ser^910^/Ser^935^ phosphorylation or 14-3-3 binding (Supplementary Figure S1 at http://www.BiochemJ.org/bj/430/bj4300405add.htm).

### Evidence that LRRK2 kinase activity controls Ser^910^ and Ser^935^ phosphorylation as well as 14-3-3 binding

**Figure 2:**
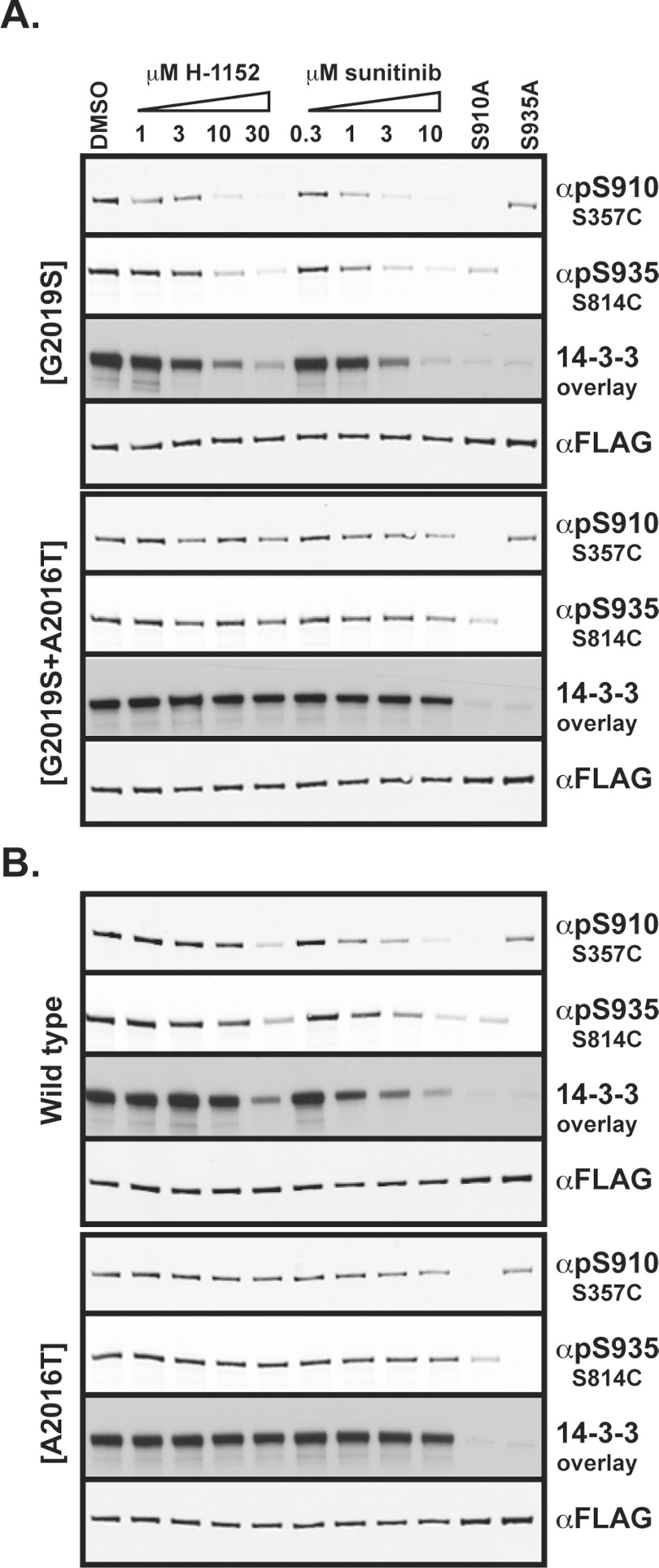
Evidence that LRRK2 kinase activity controls Ser^910^ and Ser^935^ phosphorylation, as well as 14-3-3 binding (**A** and **B**) HEK-293 cells transiently expressing the indicated forms of FLAG–LRRK2 were treated with DMSO vehicle control or indicated concentrations of H-1152 or sunitinib for 90 min. Cells were lysed in lysis buffer supplemented with 0.5% NP-40 and 150 mM NaCl and subjected to anti-FLAG immunoprecipitation. Immunoprecipitates were resolved by SDS/PAGE (4–12% Novex gels) and subjected to immunoblotting with anti-FLAG (total LRRK2), anti-phospho-Ser^910^ (αpS910), anti-phospho-Ser^935^ (αpS935) antibodies, as well as a 14-3-3 overlay assay, as indicated. Similar results were obtained in two separate experiments.

To study whether the effect of H-1152 and sunitinib on LRRK2 Ser^910^/Ser^935^ phosphorylation and 14-3-3 binding resulted from inhibition of LRRK2 protein kinase activity, we treated HEK-293 cells overexpressing LRRK2(G2019S) or the H-1152/sunitinib-resistant LRRK2(A2016T/G2019S) double-mutant with LRRK2 inhibitors. As observed with the endogenous LRRK2, we found that H-1152 and sunitinib induced a dose-dependent dephosphorylation of the Parkinson's disease LRRK2(G2019S) mutant at Ser^910^ and Ser^935^, as well as disrupting binding to 14-3-3 (Figure 2A, upper panel). Crucially, however, neither H-1152 nor sunitinib significantly inhibited Ser^910^ or Ser^935^ phosphorylation or 14-3-3 binding to the drug-resistant LRRK2(A2016T/G2019S) mutant (Figure 2A, lower panel). This strongly suggests that the ability of H-1152 and sunitinib to induce dephosphorylation of Ser^910^, as well as Ser^935^, and hence disrupt 14-3-3 binding is dependent upon the ability of these compounds to inhibit LRRK2 protein kinase activity.

In agreement with the pharmacological data demonstrating that H-1152 and sunitinib inhibit mutant LRRK2(G2019S) 2–4-fold more potently than wild-type LRRK2 [8], we found that H-1152 and sunitinib were more potent in inducing dephosphorylation and impairing binding of 14-3-3 to LRRK2(G2019S) than wild-type LRRK2 (compare the upper panels of Figures 2A and 2B). The potency of H-1152 and sunitinib at inducing dephosphorylation of wild-type FLAG–LRRK2 in HEK-293 cells was similar to the effects of these drugs on endogenous LRRK2 in Swiss 3T3 cells (compare Figures 1 and 2B).

### Analysis of LRRK2 phosphorylation in Parkinson's disease patient-derived lymphoblastoid cells

**Figure 3:**
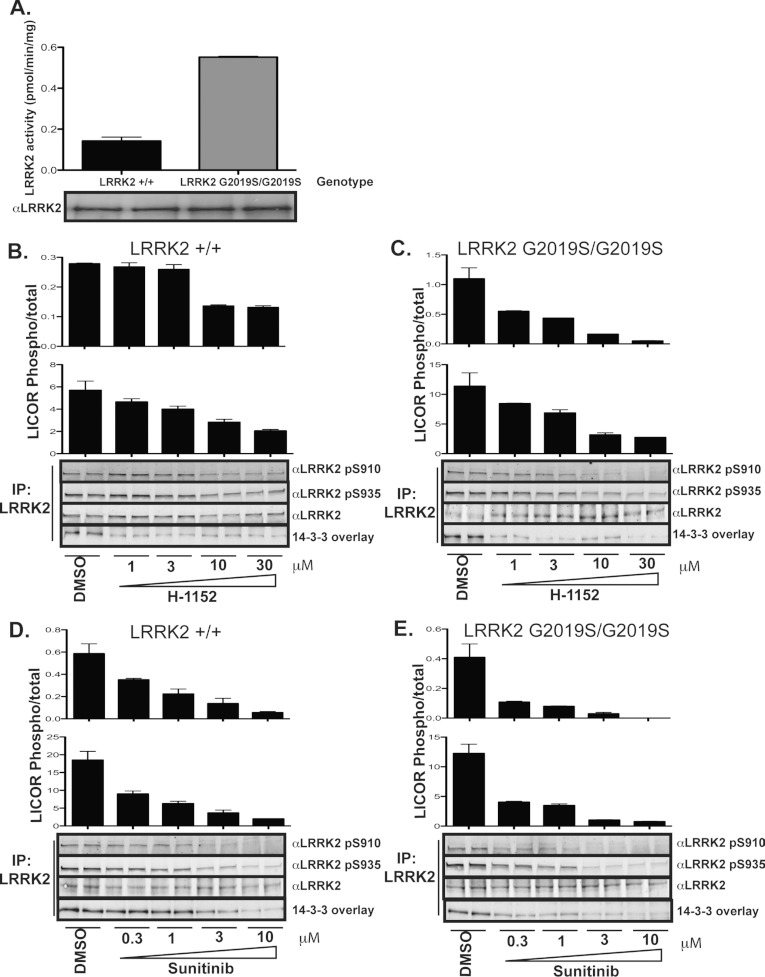
Analysis of endogenous LRRK2 Ser^910^ and Ser^935^ responsiveness to LRRK2 inhibition in Parkinson's disease patient-derived samples (**A**) LRRK2 kinase activity was assessed in EBV-immortalized lymphoblastoid cells from a patient homozygous for the LRRK2(G2019S) mutation and diagnosed with Parkinson's disease and an individual without the G2019S mutation. Kinase assays were performed in triplicate and results are means±S.E.M. (**B**–**E**) These cells were then treated with increasing doses of (**B** and **C**) H-1152 or (**D** and **E**) increasing doses of sunitinib for 60 min prior to lysis. LRRK2 was immunoprecipitated (IP) and the phosphorylation status of Ser^910^ and Ser^935^ was assessed following immunoblot and LI-COR quantification analysis. The upper histogram in each panel represents the ratio of Ser^910^ phosphorylation to total LRRK2, the lower histogram represents the ratio of Ser^935^ phosphorylation to total LRRK2. Results are means±S.E.M. for a duplicate analysis. Similar results were obtained in two separate experiments.

We also studied endogenous LRRK2 activity and phosphorylation in EBV-transformed lymphoblastoid cells derived from a Parkinson's disease patient harbouring a homozygous LRRK2(G2019S) mutation, in comparison with those derived from a second individual without an LRRK2 mutation, who presented with no disease (Figure 3). This revealed that both cell lines expressed similar levels of LRRK2 protein, but the intrinsic kinase activity of LRRK2 immunoprecipitated from the homozygous LRRK2(G2019S) cell line was approx. 3-fold higher than that observed in the wild-type cells, consistent with the G2019S mutation enhancing LRRK2 kinase activity (Figure 3A). Both wild-type LRRK2 (Figures 3B and 3D) and LRRK2(G2019S) (Figures 3C and 3E) were similarly phosphorylated at Ser^910^ and Ser^935^. H-1152 and sunitinib induced marked dephosphorylation of Ser^910^ and Ser^935^ in both wild-type and LRRK2(G2019S) human lymphoblastoid cell lines. However, consistent with the increased sensitivity of LRRK2(G2019S) for these drugs we observed that H-1152 and sunitinib inhibited phosphorylation of Ser^910^ and Ser^935^ in the LRRK2(G2019S) cells more potently than in the wild-type cells (compare Figures 3B with 3C, and 3D with 3E).

### Disruption of 14-3-3 binding alters cellular localization of LRRK2

**Figure 4:**
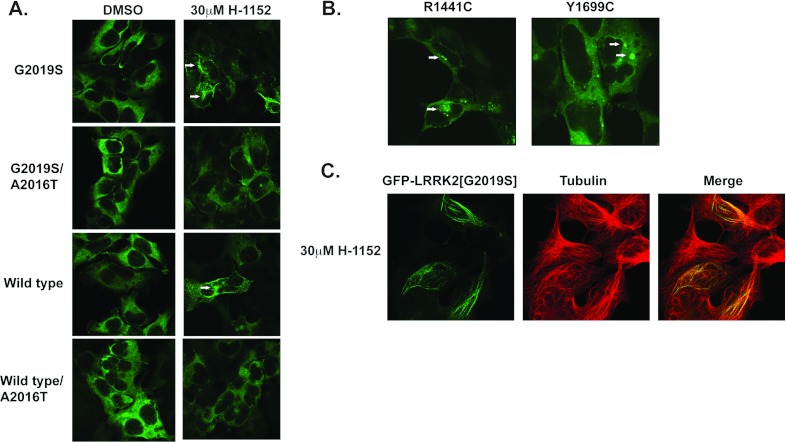
Disruption of 14-3-3 binding induces accumulation of LRRK2 within cytoplasmic aggregates (**A**) Stable-inducible T-REx cells lines harbouring the indicated forms of LRRK2 were incubated for 24 h with 0.1 μg/ml doxycycline to induce expression of GFP–LRRK2. The indicated cell lines were treated in the absence or presence of the indicated dose of H-1152 for 90 min prior to fixation. Representative fluorescent micrographs of GFP–LRRK2 localization are shown. Cytoplasmic aggregates of GFP–LRRK2 are indicated with white arrows. (**B**) Fluorescent micrographs representative of cultures of the indicated forms of GFP–LRRK2 are shown. Cytoplasmic aggregates of GFP–LRRK2 are indicated with white arrows. Localization analyses were performed in duplicate, and similar results were observed in two independent experiments. (**C**) Cells were treated as in (**A**) except that slides were co-stained with an anti-tubulin antibody to detect microtubules (red). GFP–LRRK2 is shown in green and co-localization is shown in yellow.

Mutants of LRRK2 that do not interact with 14-3-3, rather than being diffusely localized throughout the cytoplasm, accumulate within cytoplasmic aggregates [6]. This prompted us to investigate whether H-1152 treatment induced cytoplasmic re-localization of GFP–LRRK2 or GFP–LRRK2(G2019S) to discrete cytoplasmic pools (Figure 4). We employed stable inducible T-REx cells lines expressing, at low levels, drug-sensitive or drug-resistant (i.e. A2016T mutant) forms of GFP–LRRK2 or GFP–LRRK2(G2019S). As reported previously [6,16], in untreated cells GFP–LRRK2 and GFP–LRRK2(G2019S) was diffusely localized throughout the cytoplasm and not observed in the nucleus (Figure 4A). However, H-1152 treatment induced a marked accumulation of LRRK2 within cytoplasmic pools (Figure 4A). In accordance with this effect of H-1152 being mediated via inhibition of LRRK2 kinase activity, no cytoplasmic pool accumulation was observed upon treatment of cells expressing drug-resistant LRRK2(A2016T) or LRRK2(A2016T/G2019S) with H-1152 (Figure 4A). As a further control, we studied the localization of GFP–LRRK2(R1441C) and GFP–LRRK2(Y1699C), which do not bind 14-3-3 and were shown previously to accumulate within cytoplasmic pools [6,16]. We confirmed that these mutants accumulated within cytoplasmic pool-like structures, similar to those observed for GFP–LRRK2 and GFP–LRRK2(G2019S) following treatment with H-1152 (compare Figures 4A and 4B). There is some difference in the appearance of the morphology of the LRRK2 cytoplasmic inclusions that forms following drug treatment for 2 h compared with the GFP–LRRK2(R1441C) and GFP–LRRK2(Y1699C) mutants expressed in HEK-293 cells for 24–36 h (compare Figures 4A and 4B). The cytoplasmic accumulations formed after 2 h of drug treatment appears more fibrillar, suggesting association with cytoskeletal elements. To investigate this we undertook co-localization studies with anti-tubulin antibodies that revealed marked co-localization between LRRK2 cytoplasmic inclusions formed following H-1152 treatment and the microtubule network (Figure 4C).

### Evidence that LRRK2 does not autophosphorylate Ser^910^ and Ser^935^

**Figure 5:**
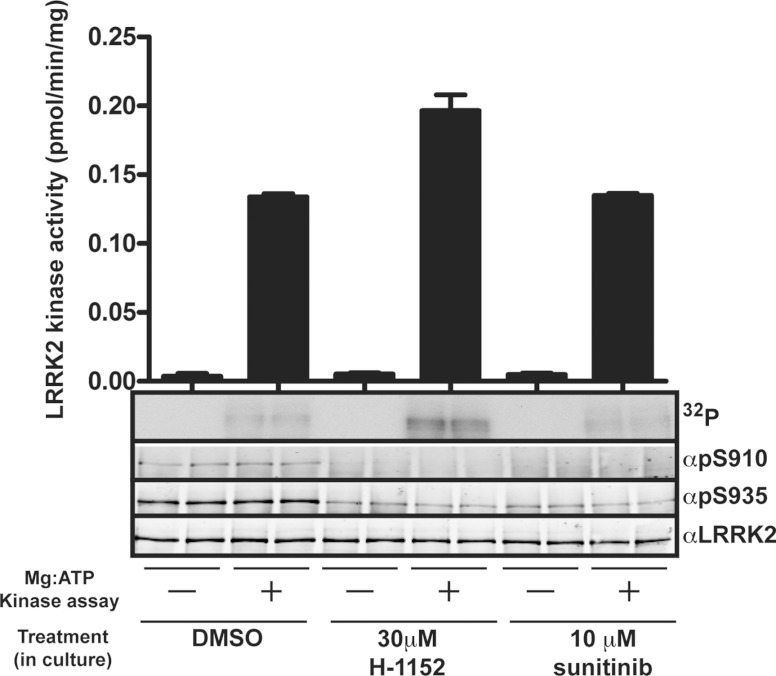
Evidence that Ser^910^/Ser^935^ phosphorylation is not mediated by autophosphorylation Endogenous LRRK2 was immunoprecipitated from Swiss 3T3 cells treated with DMSO, 30 μM H-1152 or 10 μM sunitinib for 2 h to induce dephosphorylation of Ser^910^ and Ser^935^. Immunoprecipitates were washed multiple times with lysis buffer containing 0.5 M NaCl to remove inhibitor and were then incubated in kinase buffer containing 20 μM Nictide in the presence or absence of magnesium ATP (Mg:ATP) for 30 min. Following incubation, immunoprecipitates were centrifuged at 6000 ***g*** for 0.5 min and the supernatant was spotted on to P81 paper for measurement of LRRK2 kinase activity. Sample buffer was added to the pelleted beads and LRRK2 Ser^910^ and Ser^935^ phosphorylation was quantified following immunoblot analysis with the indicated antibodies. A membrane was also subjected to autoradiography to assess LRRK2 autophosphorylation (^32^P). The minor effect that H-1152 had on LRRK2 kinase assay is not significant.

To investigate whether endogenous LRRK2 can phosphorylate itself at Ser^910^ and Ser^935^, we treated Swiss 3T3 cells with no drug, 30 μM H-1152 or 10 μM sunitinib to induce dephosphorylation of Ser^910^ and Ser^935^ (Figure 5). Endogenous LRRK2 was immunoprecipitated, and immunoprecipitates were washed to remove drug and then incubated in the absence or presence of magnesium [γ^32^P]ATP along with 20 μM LRRK2 substrate peptide for 30 min. LRRK2 kinase activity, LRRK2 autophosphorylation and phosphorylation of Ser^910^ and Ser^935^ was quantified. These studies revealed that the LRRK2 isolated from H-1152- or sunitinib-treated cells was dephosphorylated, and possessed similar activity to LRRK2 isolated from untreated cells, indicating that the drug had been efficiently removed (Figure 5). Following incubation with magnesium [γ^32^P]ATP, LRRK2 isolated from drug-treated cells autophosphorylated as judged by incorporation of ^32^P-radioactivity. Importantly, however, no increase in phosphorylation of Ser^910^ or Ser^935^ was observed following incubation of LRRK2 from H-1152-treated cells with magnesium [γ^32^P]ATP. The amount of phosphorylation of LRRK2 isolated from non-drug-treated cells on Ser^910^ and Ser^935^ was also not increased in the autophosphorylation reaction.

### Effect of multiple signal transduction inhibitors on Ser^910^/Ser^935^ phosphorylation

To gain further insight into the specificity of Ser^910^ and Ser^935^ dephosphorylation, HEK-293 cells stably expressing GFP–LRRK2 were treated with a panel of 33 kinase inhibitors including those that suppress major signal transduction pathways in cells including the PI3K (phosphoinositide 3-kinase), mTOR (mammalian target of rapamycin), ERK (extracellular-signal-regulated kinase), p38, JNK (c-Jun N-terminal kinase) and innate immune signalling pathways (Supplementary Figure S2). All inhibitors were utilized at the higher limits of concentrations known to suppress signalling pathways. As expected, 10 μM sunitinib induced marked dephosphorylation of Ser^910^ and Ser^935^, whereas 32 of the inhibitors tested did not significantly affect dephosphorylation of Ser^910^/Ser^935^. Some dephosphorylation of Ser^910^ and Ser^935^ was observed with the relatively non-specific JNK inhibitor SP600125, which was used at a concentration of 15 μM and is known to inhibit many protein kinases more potently than JNK [17]. It should be noted that the more potent JNK inhibitor AS601245 did not induce dephosphorylation of these sites. Further work will be required to delineate the protein kinase(s) that directly mediate Ser^910^ and Ser^935^ phosphorylation.

## DISCUSSION

The key finding of the present study is that the kinase activity of LRRK2 indirectly controls phosphorylation of Ser^910^/Ser^935^ and hence 14-3-3 binding, as well as LRRK2 cytoplasmic localization. In the cell lines we have investigated [Swiss 3T3 (Figure 1), HEK-293 (Figure 2) and patient-derived lymphoblastoid cells (Figure 3)] phosphorylation of LRRK2 at Ser^910^ and Ser^935^, and hence binding to 14-3-3, was reversed by treatment with the structurally diverse H-1152 and sunitinib LRRK2 inhibitors. We have also found that H-1152 induces LRRK2 to accumulate within discrete cytoplasmic pools that are similar to those observed for LRRK2 mutants that do not bind 14-3-3 [6] (Figure 4B). We conclude that dephosphorylation and cytoplasmic re-localization results from inhibition of LRRK2 kinase activity, as LRRK2 inhibitors are ineffective at inducing dephosphorylation or re-localization of drug-resistant LRRK2(A2016T) mutants (Figure 2). The finding that H-1152 and sunitinib are more potent at inducing Ser^910^/Ser^935^ dephosphorylation of LRRK2(G2019S) than wild-type LRRK2 (Figure 2), is also consistent with these drugs inhibiting LRRK2(G2019S) 2–4-fold more potently than the wild-type LRRK2 [8].

A key question concerns the mechanism by which LRRK2 controls phosphorylation of Ser^910^ and Ser^935^. One possibility is that Ser^910^ and Ser^935^ comprise direct LRRK2 autophosphorylation sites. However, our results suggest that dephosphorylated LRRK2 isolated from H-1152- or sunitinib-treated cells is unable to phosphorylate itself at Ser^910^/Ser^935^ following incubation with magnesium ATP (Figure 5). This is consistent with LRRK2 having a marked preference for phosphorylating threonine residues over serine residues, as demonstrated by the finding that replacing the phosphorylated threonine residue in an optimal peptide substrate with a serine residue abolished phosphorylation by LRRK2 [8]. Furthermore, a number of detailed studies aimed at mapping LRRK2 autophosphorylation sites have thus far not identified Ser^910^ or Ser^935^, but rather a number of phosphorylated threonine residues clustered in the LRRK2 Roc-COR domain [18–20]. A global phosphoproteomic study of a melanoma tumour identified phosphorylation of LRRK2 at Ser^935^ as one of 5600 phosphorylation sites catalogued on 2250 proteins, but this was not investigated further [21].

There is significant similarity in the sequences surrounding Ser^910^ and Ser^935^ suggesting that a single protein kinase may phosphorylate both of these residues [6]. An implication of our finding that LRRK2 inhibitors induce dephosphorylation of Ser^910^ and Ser^935^ is that the physiological Ser^910^/Ser^935^ kinase may be stimulated by LRRK2. It is also possible that LRRK2 inhibits the protein phosphatase that dephosphorylates Ser^910^ and Ser^935^. The identity of the Ser^910^/Ser^935^ kinase is unclear and thus far we have treated cells with 33 signal transduction inhibitors and found that these did not lead to dephosphorylation of Ser^910^ and Ser^935^ (Supplementary Figure S2). In future work it will be important to identify the kinase(s) and/or protein phosphatase(s) that act on Ser^910^ and Ser^935^ and to determine whether it is controlled by LRRK2.

We observed that kinase-dead LRRK2 is still partially phosphorylated at Ser^910^ and Ser^935^ when overexpressed in HEK-293 cells (Supplementary Figure S3A at http://www.BiochemJ.org/bj/430/bj4300405add.htm). It is possible that the downstream kinase that LRRK2 regulates may still be partially active in HEK-293 cells and thus capable of phosphorylating the kinase-dead LRRK2 at Ser^910^ and Ser^935^ to some extent, especially over the 24–36 h time period in which expression of LRRK2 was induced. If this were the case, we would predict that phosphorylation of kinase-dead LRRK2 at Ser^910^ and Ser^935^ would not be influenced by LRRK2 inhibitors, as we have not been able to detect significant levels of endogenous LRRK2 in HEK-293 cells [8]. Consistent with this, we find that H-1152 and sunitinib LRRK2 inhibitors do not induce dephosphorylation of kinase-dead LRRK2 at Ser^910^ and Ser^935^ (Supplementary Figure S3B). Moreover, H-1152 did not induce kinase-dead LRRK2 to accumulate within cytoplasmic pools under conditions whereas the wild-type enzyme accumulated within these pools (Supplementary Figure S3C). We have also observed that the 2-fold more active LRRK2(G2019S) mutant and wild-type LRRK2 are phosphorylated to the same extent on Ser^910^ and Ser^935^ [6]. If overexpression of wild-type LRRK2 is sufficient to maximally stimulate the downstream pathway that regulates phosphorylation of LRRK2 at Ser^910^ and Ser^935^, this would explain why the more active LRRK2(G2019S) mutant is not phosphorylated to a greater extent.

How 14-3-3 interaction influences LRRK2 function requires further investigation. Our results suggest that this interaction does not control LRRK2 protein kinase activity, as mutation of Ser^910^ and/or Ser^935^ does not influence LRRK2 catalytic activity [6]. Consistent with this, treatment of cells with H-1152 or sunitinib induced dephosphorylation of Ser^910^ and Ser^935^ as well as disrupting 14-3-3 binding, but did not affect endogenous LRRK2 kinase activity (Figure 5). However, the finding that 14-3-3 binding influences the cytoplasmic localization of LRRK2 has implications for drug therapy as it has been suggested that the cytoplasmic pools that several non-14-3-3-binding LRRK2 mutants [including LRRK2(R1441C) and LRRK2(Y1699C)] accumulate within might comprise aggregates of misfolded unstable LRRK2 protein [11]. In future work it would be important to investigate further whether disruption of 14-3-3 binding using LRRK2 inhibitors affects LRRK2 stability, as well as interaction with substrates. It would also be important to undertake more detailed characterization of the cytoplasmic pools that LRRK2 accumulates in following treatment with LRRK2 inhibitors and to determine whether these structures do indeed comprise pools of misfolded LRRK2 protein.

Our finding that all of the structurally diverse LRRK2 inhibitors tested to date induce dephosphorylation of Ser^910^ and Ser^935^ suggests that the physiological Ser^910^/Ser^935^ kinase and/or phosphatase is somehow regulated by LRRK2 and that LRRK2 might function as an upstream protein kinase. Our results suggest that phosphorylation of LRRK2 at Ser^910^ and Ser^935^ may function as a regulatory feedback network. Similar regulatory feedback loops operate in many other signal transduction pathways, where a downstream kinase phosphorylates an upstream component of the signal transduction pathway. For example, in the ERK signalling pathway ERK1/ERK2 phosphorylate the upstream MEK [MAPK (mitogen-activated protein kinase)/ERK kinase] protein kinase [22], whereas RSK (ribosomal S6 kinase) a kinase that is activated by ERK, phosphorylates and inhibits mSOS (mammalian Son of sevenless), the guanine-nucleotide-exchange factor for Ras [23]. In the PI3K pathway, S6K1 (S6 kinase 1) phosphorylates upstream components such as IRS (insulin receptor substrate) adaptors [24] and Rictor (rapamycin-insensitive companion of mTOR) [25] to regulate the pathway. These regulatory feedback loops are inherent features of signal transduction pathways and play vital roles in modulating the extent and duration of signalling networks.

**Figure 6:**
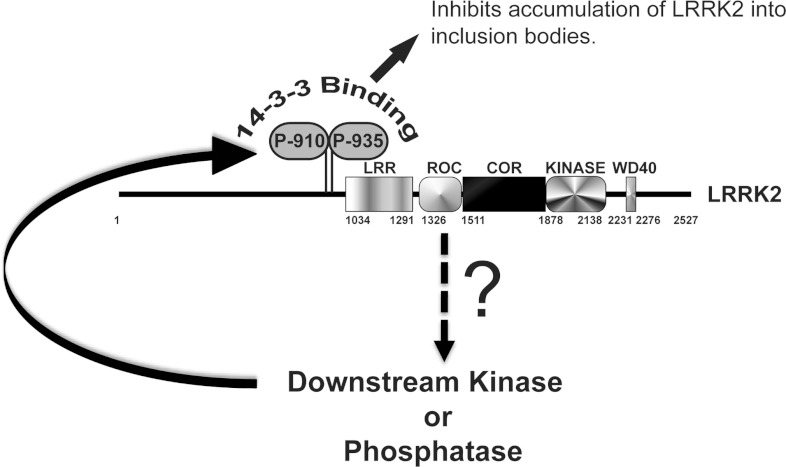
Proposed model of how LRRK2 controls Ser^910^ and Ser^935^ phosphorylation leading to 14-3-3 binding Our data suggest that LRRK2 functions as an upstream component of a signal transduction pathway that either directly or indirectly stimulates the activity of a protein kinase or inhibits the activity of a protein phosphatase that acts on Ser^910^ and Ser^935^. This enables LRRK2 to interact with 14-3-3 isoforms and stabilizes diffuse cytoplasmic localization of LRRK2. Treatment of cells with LRRK2 inhibitors thus leads to dephosphorylation of Ser^910^ and Ser^935^ and dissociation of 14-3-3. Our findings indicate that LRRK2 phosphorylation of Ser^910^ and Ser^935^ as well as 14-3-3 binding could be employed as a biomarker to benchmark efficacy of LRRK2 inhibitors that are being developed.

In Figure 6 we present a model by which phosphorylation of Ser^910^ and Ser^935^ is dependent upon LRRK2 activity and hence mediates binding to 14-3-3 isoforms. We propose that monitoring LRRK2 phosphorylation at Ser^910^/Ser^935^ and/or 14-3-3 binding, and/or cytoplasmic localization of LRRK2, can be utilized as a cell-based read-out to evaluate the relative potency of LRRK2 inhibitors. These assays could be deployed in cell lines, tissues of animals or humans treated with LRRK2 inhibitors. Our results show that LRRK2 inhibitors induced dephosphorylation and disrupted 14-3-3 binding to endogenous LRRK2 from patient-derived lymphoblastoid cell lines (Figure 3). This indicates that, for human patients administered LRRK2 inhibitors, the phosphorylation status of LRRK2 at Ser^910^ and Ser^935^ in blood cells could be employed as a biomarker of LRRK2 inhibitor activity. To our knowledge, the present paper describes the first approach that could be exploited to develop a simple cell-based system to assess the efficacy of LRRK2 protein kinase inhibitors. Our findings will also stimulate future work aimed at understanding how Ser^910^ and Ser^935^ phosphorylation is regulated by protein kinase(s) or phosphatase(s) and the role that LRRK2 plays in influencing feedback phosphorylation of these residues. It would be fascinating to explore whether the Ser^910^/Ser^935^ protein kinase and/or phosphatase enzymes might also comprise a drug target for the treatment of Parkinson's disease. Finally, it will be vital to work out how 14-3-3 affects LRRK2 function and whether this is relevant to understanding the role of LRRK2 in Parkinson's disease.

## Online data

Supplementary data
