# Supplementary material for: Inhibition of LRRK2 kinase activity leads to dephosphorylation of Ser910/Ser935, disruption of 14-3-3 binding and altered cytoplasmic localization
Source: Biochem J. 2010 Aug 27;430(Pt 3):405–13. doi: 10.1042/BJ20100784 (PMC3631100; doi:10.1042/BJ20100784)
Supplement: Supplementary data [file bj4300405add.pdf]

BJ www.biochemj.org

BJ ChemBio

Biochem. J. (2010) **430**, 405–413 (Printed in Great Britain) doi:10.1042/BJ20100784

online data

SUPPLEMENTARY ONLINE DATA

Inhibition of LRRK2 kinase activity leads to dephosphorylation of Ser^{910}/Ser^{935}, disruption of 14-3-3 binding and altered cytoplasmic localization

Nicolas DZAMKO*, Maria DEAK*, Faycal HENTATI†, Alastair D. REITH‡, Alan R. PRESCOTT§, Dario R. ALESSI*[1] and R. Jeremy NICHOLS*[1,2]

*MRC Protein Phosphorylation Unit, College of Life Sciences, University of Dundee, Dow Street, Dundee DD1 5EH, Scotland, U.K., †Department of Neurology, Institut National de Neurologie, Tunis, Tunisia, ‡External Alliances & Development, GlaxoSmithKline (China) R&D Co. Ltd, Medicines Research Centre, Gunnels Wood Road, Stevenage SG1 2NY, U.K., and §Division of Cell Biology and Immunology, College of Life Sciences, University of Dundee, Dow Street, Dundee DD1 5EH, Scotland, U.K.

Biochemical Journal

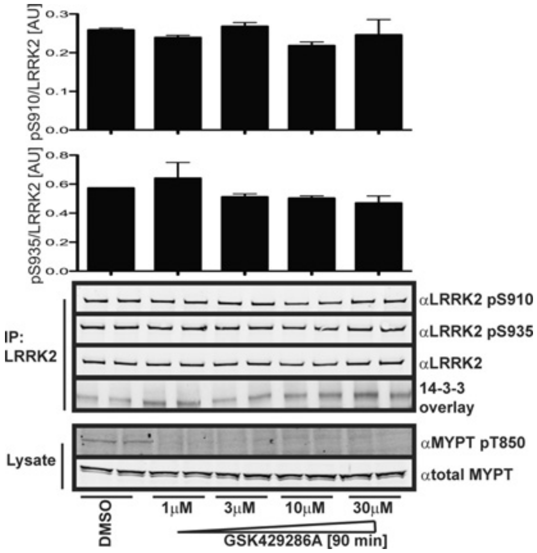

Figure S1 Phosphorylation of LRRK2 at Ser^{910} and Ser^{935} is not regulated by ROCK

Endogenous LRRK2 was immunoprecipitated (IP) with anti-LRRK2 100–500 (S348C) antibody from Swiss 3T3 cells treated with DMSO vehicle control or the indicated concentrations of the potent ROCK inhibitor GSK429286A for 90 min. Immunoprecipitates were subjected to immunoblot analysis with the indicated antibody as well as 14-3-3 overlay far-Western analysis. The immunoblot analysis was quantified by Odyssey® LI-COR analysis and the amount of LRRK2 phosphorylation is presented as the ratio of phosphospecific antibody to total LI-COR (phospho-Ser^{910}/LRRK2) in absorbance units (AU).

[1] Correspondence may be addressed to either of these authors (email d.r.alessi@dundee.ac.uk or jnichols@parkinsonsinstitute.org).
[2] Present address: Parkinson's Institute, 675 Almanor Avenue, Sunnyvale, CA 94085, U.S.A.

© 2013 The Author(s)
The author(s) has paid for this article to be freely available under the terms of the Creative Commons Attribution Licence (CC-BY) (http://creativecommons.org/licenses/by/3.0/) which permits unrestricted use, distribution and reproduction in any medium, provided the original work is properly cited.

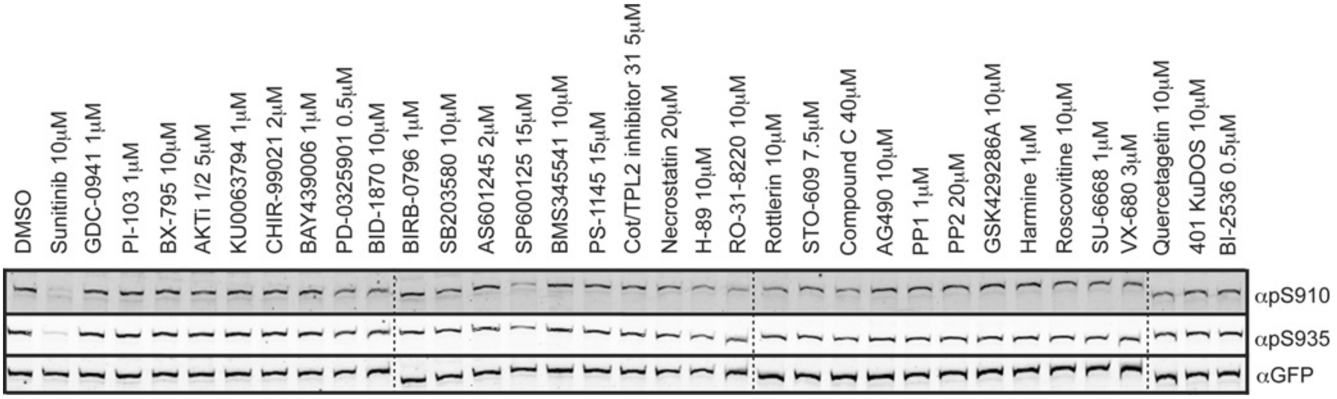

Figure S2 Effect of 33 signal transduction inhibitors on LRRK2 Ser^{910} and Ser^{935} phosphorylation

HEK-293 cells stably expressing GFP–LRRK2 were treated with DMSO, or the following inhibitors dissolved in DMSO, at the indicated concentration for 90 min: sunitinib (LRRK2 inhibitor [1]); GDC-0941 (PI3K inhibitor [2]); PI-103 (dual mTOR/PI3K inhibitor [3]); BX-795 {dual MARK/PDK1 (phosphoinositide-dependent kinase 1) inhibitor [4]}; AKTi1/2 {PKB (protein kinase B) inhibitor [5]}; KU0063794 (mTOR inhibitor [6]); CHIR-99021 {GSK3 (glycogen synthase kinase 3) inhibitor [7]}; BAY439006 (Raf inhibitor [8]); PD-0325901 (MEK1 inhibitor [9]); BID-1870 (RSK inhibitor [10]); BIRB-0796 (p38 MAPK inhibitor [11]); SB203580 (p38 MAPK inhibitor [12]); AS601245 (JNK inhibitor [13]); SP600125 (JNK inhibitor [14]); BMS345541 {IKK (inhibitor of nuclear factor κB kinase) inhibitor [15]}; PS-1145 (IKK inhibitor [16]); TPL2 inhibitor 31 {Cot/TPL2 (tumour progression locus 2) inhibitor [17]}; Necrostatin {RIPK (receptor-interacting serine/threonine protein kinase) inhibitor [18]}; H-89 {dual PKA (protein kinase A) /ROCK inhibitor [19]}; RO-31-8220 {PKC (protein kinase A) inhibitor [20]}; Rottlerin (PKC inhibitor [21]); STO-609 {CaMKK (calcium/calmodulin-dependent protein kinase kinase) inhibitor [22]}; Compound C {AMPK (AMP-activated protein kinase) inhibitor [23]}; AG490 {JAK (Janus kinase) inhibitor [24]}; PP1 (Src inhibitor [25]); PP2 (Src inhibitor [26]); GSK429286A (ROCK inhibitor [1]); Harmine (dual CDK (cyclin-dependent kinase)/DYRK (dual-specificity tyrosine-phosphorylated and -regulated kinase) inhibitor [27]); Roscovitine (CDK inhibitor [28]); SU-6668 (dual Src/Aurora kinase inhibitor [29]); VX-680 (Aurora kinase inhibitor [30]); Quercetagetin {PIMK (Pim kinase) inhibitor [31]}; 401 KuDOS {DNAPK (DNA-dependent protein kinase) inhibitor [32]}; and BI-2536 {PLK1 (Polo-like kinase 1) inhibitor [33]}. Following lysis, 30 μg of lysate was resolved by SDS/PAGE and immunoblotted for LRRK2 phosphorylation at Ser^{910} and Ser^{935}. Total LRRK2 was assessed by anti-GFP antibody immunoblotting. The immunoblots shown are representative of two independent experiments.

© 2013 The Author(s)
The author(s) has paid for this article to be freely available under the terms of the Creative Commons Attribution Licence (CC-BY) (http://creativecommons.org/licenses/by/3.0/) which permits unrestricted use, distribution and reproduction in any medium, provided the original work is properly cited.

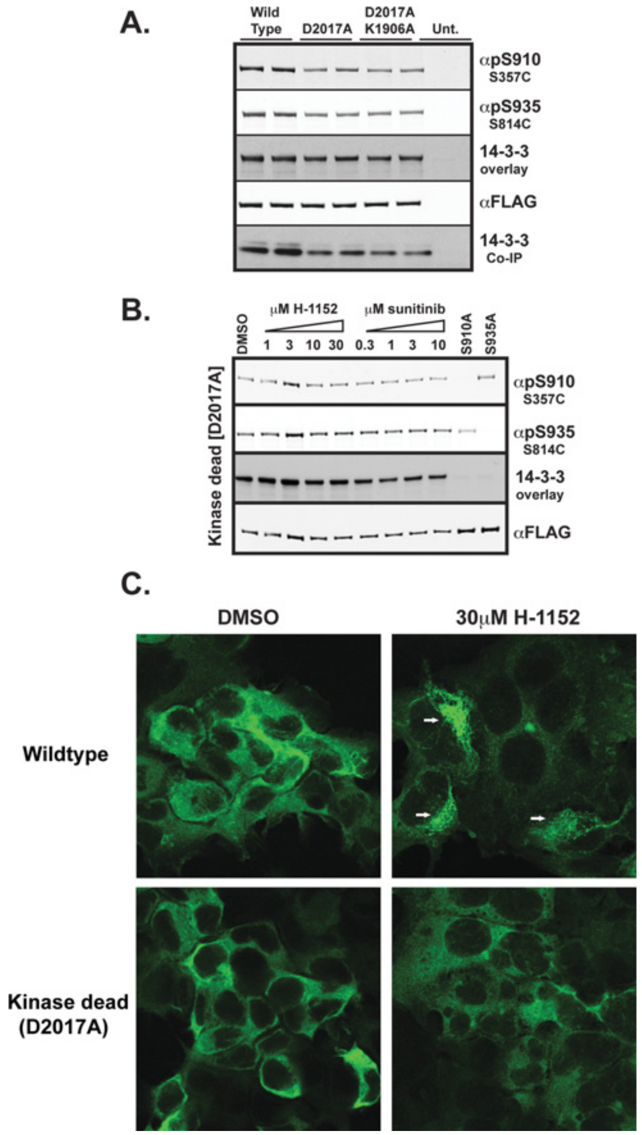

Figure S3 Analysis of Ser^{910} and Ser^{935} phosphorylation in kinase-inactive mutants of LRRK2

(**A**) FLAG-tagged wild-type LRRK2 and the indicated kinase-inactive versions, LRRK2(D2017A) and a double kinase-inactive LRRK2(K1906A/D2017A) mutant (that should be completely devoid of even trace levels of kinase activity), were transiently expressed in HEK-293 cells and immunoprecipitated with anti-FLAG beads. Immunoprecipitates were analysed by immunoblot analysis with anti-FLAG (for total LRRK2), anti-phospho-Ser^{910} (αpS910) and anti-phospho-Ser^{935} (αpS935) antibodies as well as a 14-3-3 overlay assay. Co-immunoprecipitated 14-3-3 was assessed by immunoblotting with the anti-pan-14-3-3 antibody. (**B**) HEK-293 cells transiently expressing FLAG–LRRK2(D2017A) were treated with DMSO vehicle control or the indicated concentrations of H-1152 or sunitinib for 90 min. Cells were lysed in lysis buffer supplemented with 0.5% NP-40 and 150 mM NaCl and subjected to anti-FLAG antibody immunoprecipitation. Immunoprecipitates were resolved by SDS/PAGE (4–12 % Novex gels) and subjected to immunoblotting with anti-FLAG (for total LRRK2), anti-phospho-Ser^{910} (αpS910) and anti-phospho-Ser^{935} (αpS935) antibodies, as well as a 14-3-3 overlay assay. (**C**) Stable-inducible T-REx cells lines harbouring the indicated forms of LRRK2 were induced for 24 h with 0.1 μg/ml doxycycline to induce expression of GFP–LRRK2. The indicated cell lines were treated in the absence or presence of the indicated dose of H-1152 for 90 min prior to fixation. Representative fluorescent micrographs of GFP–LRRK2 localization are shown. Cytoplasmic aggregates resembling inclusion bodies of GFP–LRRK2 are indicated with white arrows.

REFERENCES

1 Nichols, R. J., Dzamko, N., Hutti, J. E., Cantley, L. C., Deak, M., Moran, J., Bamborough, P., Reith, A. D. and Alessi, D. R. (2009) Substrate specificity and inhibitors of LRRK2, a protein kinase mutated in Parkinson's disease. Biochem. J. **424**, 47–60

2 Raynaud, F. I., Eccles, S. A., Patel, S., Alix, S., Box, G., Chuckowree, I., Folkes, A., Gowan, S., De Haven Brandon, A., Di Stefano, F. et al. (2009) Biological properties of potent inhibitors of class I phosphatidylinositide 3-kinases: from PI-103 through PI-540, PI-620 to the oral agent GDC-0941. Mol. Cancer Ther. **8**, 1725–1738

3 Fan, Q. W., Knight, Z. A., Goldenberg, D. D., Yu, W., Mostov, K. E., Stokoe, D., Shokat, K. M. and Weiss, W. A. (2006) A dual PI3 kinase/mTOR inhibitor reveals emergent efficacy in glioma. Cancer Cell **9**, 341–349

4 Feldman, R. I., Wu, J. M., Polokoff, M. A., Kochanny, M. J., Dinter, H., Zhu, D., Biroc, S. L., Alicke, B., Bryant, J., Yuan, S. et al. (2005) Novel small molecule inhibitors of 3-phosphoinositide-dependent kinase-1. J. Biol. Chem. **280**, 19867–19874

5 Logie, L., Ruiz-Alcaraz, A. J., Keane, M., Woods, Y. L., Bain, J., Marquez, R., Alessi, D. R. and Sutherland, C. (2007) Characterization of a protein kinase B inhibitor *in vitro* and in insulin-treated liver cells. Diabetes **56**, 2218–2227

6 Garcia-Martinez, J. M., Moran, J., Clarke, R. G., Gray, A., Cosulich, S. C., Chresta, C. M. and Alessi, D. R. (2009) Ku-0063794 is a specific inhibitor of the mammalian target of rapamycin (mTOR). Biochem. J. **421**, 29–42

7 Ring, D. B., Johnson, K. W., Henriksen, E. J., Nuss, J. M., Goff, D., Kinnick, T. R., Ma, S. T., Reeder, J. W., Samuels, I., Slabiak, T. et al. (2003) Selective glycogen synthase kinase 3 inhibitors potentiate insulin activation of glucose transport and utilization *in vitro* and *in vivo*. Diabetes **52**, 588–595

8 Wilhelm, S. M., Carter, C., Tang, L., Wilkie, D., McNabola, A., Rong, H., Chen, C., Zhang, X., Vincent, P., McHugh, M. et al. (2004) BAY 43-9006 exhibits broad spectrum oral antitumor activity and targets the RAF/MEK/ERK pathway and receptor tyrosine kinases involved in tumor progression and angiogenesis. Cancer Res. **64**, 7099–7109

9 Barrett, S. D., Bridges, A. J., Dudley, D. T., Saltiel, A. R., Fergus, J. H., Flamme, C. M., Delaney, A. M., Kaufman, M., LePage, S., Leopold, W. R. et al. (2008) The discovery of the benzhydroxamate MEK inhibitors CI-1040 and PD 0325901. Bioorg. Med. Chem. Lett. **18**, 6501–6504

10 Sapkota, G. P., Cummings, L., Newell, F. S., Armstrong, C., Bain, J., Frodin, M., Grauert, M., Hoffmann, M., Schnapp, G., Steegmaier, M. et al. (2007) BI-D1870 is a specific inhibitor of the p90 RSK (ribosomal S6 kinase) isoforms *in vitro* and *in vivo*. Biochem. J. **401**, 29–38

11 Pargellis, C., Tong, L., Churchill, L., Cirillo, P. F., Gilmore, T., Graham, A. G., Grob, P. M., Hickey, E. R., Moss, N., Pav, S. and Regan, J. (2002) Inhibition of p38 MAP kinase by utilizing a novel allosteric binding site. Nat. Struct. Biol. **9**, 268–272

12 Cuenda, A., Rouse, J., Doza, Y. N., Meier, R., Cohen, P., Gallagher, T. F., Young, P. R. and Lee, J. C. (1995) SB 203580 is a specific inhibitor of a MAP kinase homologue which is stimulated by cellular stresses and interleukin-1. FEBS Lett. **364**, 229–233

13 Gaillard, P., Jeanclaude-Etter, I., Ardissone, V., Arkinstall, S., Cambet, Y., Camps, M., Chabert, C., Church, D., Cirillo, R., Gretener, D. et al. (2005) Design and synthesis of the first generation of novel potent, selective, and *in vivo* active (benzothiazol-2-yl)acetonitrile inhibitors of the c-Jun N-terminal kinase. J. Med. Chem. **48**, 4596–4607

14 Bennett, B. L., Sasaki, D. T., Murray, B. W., O'Leary, E. C., Sakata, S. T., Xu, W., Leisten, J. C., Motiwala, A., Pierce, S., Satoh, Y. et al. (2001) SP600125, an anthrapyrazolone inhibitor of Jun N-terminal kinase. Proc. Natl. Acad. Sci. U.S.A. **98**, 13681–13686

15 Burke, J. R., Pattoli, M. A., Gregor, K. R., Brassil, P. J., MacMaster, J. F., McIntyre, K. W., Yang, X., Iotzova, V. S., Clarke, W., Strnad, J. et al. (2003) BMS-345541 is a highly selective inhibitor of IκB kinase that binds at an allosteric site of the enzyme and blocks NF-κB-dependent transcription in mice. J. Biol. Chem. **278**, 1450–1456

16 Castro, A. C., Dang, L. C., Soucy, F., Grenier, L., Mazdiyasni, H., Hottelet, M., Parent, L., Pien, C., Palombella, V. and Adams, J. (2003) Novel IKK inhibitors: β-carbolines. Bioorg. Med. Chem. Lett. **13**, 2419–2422

17 Hu, Y., Green, N., Gavrin, L. K., Janz, K., Kaila, N., Li, H. Q., Thomason, J. R., Cuozzo, J. W., Hall, J. P., Hsu, S. et al. (2006) Inhibition of Tpl2 kinase and TNFα production with quinoline-3-carbonitriles for the treatment of rheumatoid arthritis. Bioorg. Med. Chem. Lett. **16**, 6067–6072

18 Degterev, A., Hitomi, J., Germscheid, M., Ch'en, I. L., Korkina, O., Teng, X., Abbott, D., Cuny, G. D., Yuan, C., Wagner, G. et al. (2008) Identification of RIP1 kinase as a specific cellular target of necrostatins. Nat. Chem. Biol. **4**, 313–321

19 Leemhuis, J., Boutillier, S., Schmidt, G. and Meyer, D. K. (2002) The protein kinase A inhibitor H89 acts on cell morphology by inhibiting Rho kinase. J. Pharmacol. Exp. Ther. **300**, 1000–1007

20 Muid, R. E., Dale, M. M., Davis, P. D., Elliott, L. H., Hill, C. H., Kumar, H., Lawton, G., Twomey, B. M., Wadsworth, J., Wilkinson, S. E. et al. (1991) A novel conformationally restricted protein kinase C inhibitor, Ro 31-8425, inhibits human neutrophil superoxide generation by soluble, particulate and post-receptor stimuli. FEBS Lett. **293**, 169–172

© 2013 The Author(s)
The author(s) has paid for this article to be freely available under the terms of the Creative Commons Attribution Licence (CC-BY) (http://creativecommons.org/licenses/by/3.0/) which permits unrestricted use, distribution and reproduction in any medium, provided the original work is properly cited.

21 Gschwendt, M., Muller, H. J., Kielbassa, K., Zang, R., Kittstein, W., Rincke, G. and Marks, F. (1994) Rottlerin, a novel protein kinase inhibitor. Biochem. Biophys. Res. Commun. **199**, 93–98
22 Tokumitsu, H., Inuzuka, H., Ishikawa, Y. and Kobayashi, R. (2003) A single amino acid difference between α and β Ca^{2+}/calmodulin-dependent protein kinase kinase dictates sensitivity to the specific inhibitor, STO-609. J. Biol. Chem. **278**, 10908–10913
23 Zhou, G., Myers, R., Li, Y., Chen, Y., Shen, X., Fenyk-Melody, J., Wu, M., Ventre, J., Doebber, T., Fujii, N. et al. (2001) Role of AMP-activated protein kinase in mechanism of metformin action. J. Clin. Invest. **108**, 1167–1174
24 Meydan, N., Grunberger, T., Dadi, H., Shahar, M., Arpaia, E., Lapidot, Z., Leeder, J. S., Freedman, M., Cohen, A., Gazit, A. et al. (1996) Inhibition of acute lymphoblastic leukaemia by a Jak-2 inhibitor. Nature **379**, 645–648
25 Hanke, J. H., Gardner, J. P., Dow, R. L., Changelian, P. S., Brissette, W. H., Weringer, E. J., Pollok, B. A. and Connelly, P. A. (1996) Discovery of a novel, potent, and Src family-selective tyrosine kinase inhibitor. Study of Lck- and FynT-dependent T cell activation. J. Biol. Chem. **271**, 695–701
26 Nagao, M., Kaziro, Y. and Itoh, H. (1999) The Src family tyrosine kinase is involved in Rho-dependent activation of c-Jun N-terminal kinase by Gα12. Oncogene **18**, 4425–4434
27 Song, Y., Kesuma, D., Wang, J., Deng, Y., Duan, J., Wang, J. H. and Qi, R. Z. (2004) Specific inhibition of cyclin-dependent kinases and cell proliferation by harmine. Biochem. Biophys. Res. Commun. **317**, 128–132

Received 28 May 2010/16 July 2010; accepted 21 July 2010
Published as BJ Immediate Publication 21 July 2010, doi:10.1042/BJ20100784

28 Meijer, L., Borgne, A., Mulner, O., Chong, J. P., Blow, J. J., Inagaki, N., Inagaki, M., Delcros, J. G. and Moulinoux, J. P. (1997) Biochemical and cellular effects of roscovitine, a potent and selective inhibitor of the cyclin-dependent kinases cdc2, cdk2 and cdk5. Eur. J. Biochem. **243**, 527–536
29 Blake, R. A., Broome, M. A., Liu, X., Wu, J., Gishizky, M., Sun, L. and Courtneidge, S. A. (2000) SU6656, a selective src family kinase inhibitor, used to probe growth factor signaling. Mol. Cell. Biol. **20**, 9018–9027
30 Harrington, E. A., Bebbington, D., Moore, J., Rasmussen, R. K., Ajose-Adeogun, A. O., Nakayama, T., Graham, J. A., Demur, C., Hercend, T., Diu-Hercend, A. et al. (2004) VX-680, a potent and selective small-molecule inhibitor of the Aurora kinases, suppresses tumor growth *in vivo*. Nat. Med. **10**, 262–267
31 Holder, S., Zemskova, M., Zhang, C., Tabrizizad, M., Bremer, R., Neidigh, J. W. and Lilly, M. B. (2007) Characterization of a potent and selective small-molecule inhibitor of the PIM1 kinase. Mol. Cancer Ther. **6**, 163–172
32 Hardcastle, I. R., Cockcroft, X., Curtin, N. J., El-Murr, M. D., Leahy, J. J., Stockley, M., Golding, B. T., Rigoreau, L., Richardson, C., Smith, G. C. and Griffin, R. J. (2005) Discovery of potent chromen-4-one inhibitors of the DNA-dependent protein kinase (DNA-PK) using a small-molecule library approach. J. Med. Chem. **48**, 7829–7846
33 Steegmaier, M., Hoffmann, M., Baum, A., Lenart, P., Petronczki, M., Krssak, M., Gurtler, U., Garin-Chesa, P., Lieb, S., Quant, J. et al. (2007) BI 2536, a potent and selective inhibitor of polo-like kinase 1, inhibits tumor growth *in vivo*. Curr. Biol. **17**, 316–322

© 2013 The Author(s)
The author(s) has paid for this article to be freely available under the terms of the Creative Commons Attribution Licence (CC-BY) (http://creativecommons.org/licenses/by/3.0/) which permits unrestricted use, distribution and reproduction in any medium, provided the original work is properly cited.
